# IDH1/2 Mutants Inhibit TET-Promoted Oxidation of RNA 5mC to 5hmC

**DOI:** 10.1371/journal.pone.0161261

**Published:** 2016-08-22

**Authors:** Qiang Xu, Kai Wang, Lina Wang, Yuting Zhu, Guangyu Zhou, Dan Xie, Qingkai Yang

**Affiliations:** Department of Oncology, Second Affiliated Hospital, Institute of Cancer Stem Cell, DaLian Medical University, 9 Western Lvshun South Road, Dalian, Liaoning 116044, China; New England BioLabs Inc, UNITED STATES

## Abstract

TETs (TET1/2/3) play critical roles in multi cellular processes through DNA demethylation driven by oxidation of DNA 5mdC to 5hmdC. Interestingly, recent studies indicated that TETs also oxidate RNA 5mC to 5hmC. However, little is known about the distribution of RNA 5hmC and the regulatory mechanism of RNA 5hmC in human. Here, we show that 5hmC is enriched in mRNA, and IDH1/2 mutants inhibit TET-promoted oxidation of RNA 5mC to 5hmC. Since IDH1/2 mutations have been described to block the DNA oxidative activity of TETs, we hypothesized that IDH1/2 mutations might also inhibit the RNA oxidative activity of TETs. To evaluate the role of IDH1/2 mutations in RNA 5hmC, TETs with/without IDH1/2 mutants were overexpressed in human HEK293 cells. Resultant DNA and RNA were digested and analyzed by triple-quadrupole LC mass spectrometer. DNA 5hmdC and RNA 5hmC modifications were quantified with external calibration curves of appropriate standards. It was found that compared with total RNA (5hmC/C: less than 2 X 10^−7^), mRNA showed much higher 5hmC level (5hmC/C: ∼7 X 10^−6^). Further study indicated that IDH1/2 mutants showed significant ability to inhibit TET-promoted RNA5hmC. Consistent with this result, overexpression of IDH1/2 mutants also inhibited TET catalytic domain-promoted oxidation of RNA. In this study, we show not only the enrichment of 5hmC in mRNA, but also a regulatory mechanism of RNA 5hmC—IDH1/2 mutations inhibit TET-promoted RNA 5hmC, which suggests an involvement of IDH1/2 mutations in tumorigenesis through the deregulation of RNA biology.

## Introduction

**Fig 1:**
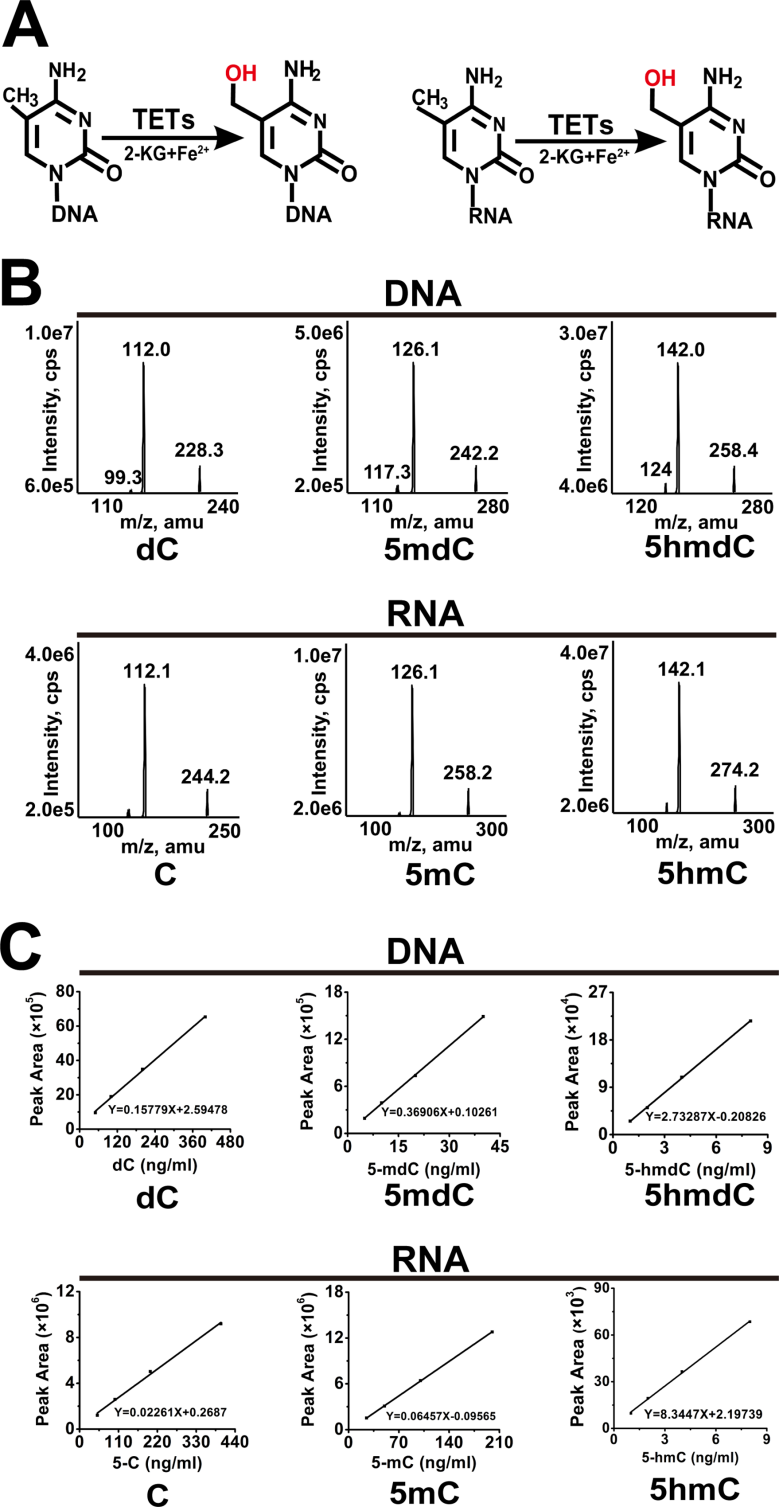
Triple quadrupole mass spectrometer was applied to assess DNA and RNA modifications. (A) Scheme of oxidation of DNA and RNA by TETs. (B) Base ion mass transitions for LC-MS/MS analysis of DNA nucleosides (dC, 5mdC and 5hmdC) and RNA nucleosides (C, 5mC and 5hmC) as noted. The nucleosides(C, 5mC, 5hmC,dC, 5mdC and 5hmdC) were separated by reverse phase ultra-performance liquid chromatography on a C18 column, with online mass spectrometry detection using triple-quadrupole LC mass spectrometer in positive electrospray ionization mode. The nucleosides were quantified using the nucleoside to base ion mass transitions of 228 to 112 (dC), 242 to 126 (5mdC), 258 to 142 (5hmdC), 244 to 112 (C), 258 to 126 (5mC), and 274 to 142 (5hmC). More details are described in Methods and Materials. (C) DNA (dC, 5mdC and 5hmdC) and RNA (C, 5mC and 5hmC) nucleoside standard curves as noted. Standard curves were built by serial dilutions. Y-axis: normalized peak area of nucleoside standards as noted; X-axis: concentration of nucleoside standards as noted. More details are described in Methods and Materials.

There are three members in ten-eleven translocation (TET) family in human, namely TET1, TET2 and TET3. Facilitated by 2-ketoglutarate (2-KG) and Fe^2+^, TETs (TET1/2/3) have been reported to sequentially oxidate 5-methyl-2’-deoxycytidine (5mdC) first to 5-hydroxymethyl-2’-deoxycytidine (5hmdC) (Fig 1A, left panel), then to 5-formyl-2’-deoxycytidine (5fdC), and finally to 5-carboxyl-2’-deoxycytidine (5cadC) [1–3]. Consequently, facilitated by DNA repair enzymes such as thymine-DNA glycosylase (TDG), TETs promote DNA oxidation and result in DNA demethylation [4]. Many studies have indicated that TETs play important roles in various physical and pathological processes, including tumorigenesis [5], cell reprogramming [6, 7], development [8], and differentiation [9, 10]. As the most frequently mutated member of TET family, TET2 is often mutated in both lymphoid malignancies (such as T cell lymphoma 30%–40%) and myeloid malignancies, especially acute myeloid leukemia (AML, 7%–23%) [11, 12]. In the meantime, previous studies showed that decrease of TETs and 5hmdC is identified as a hallmark of multi kinds of solid tumors[13, 14], whereas enhanced expression of TET2 suppresses theproliferation and metastasis of cancer cells [13, 15]. Although it is generally believed that TETs play critical roles in cellular processes through DNA oxidation, recent studies indicated that TET ortholog (CG43444) and TETs are also able to oxidate RNA 5-methylcytidine (5mC) to 5-hydroxymethylcytidine (5hmC) [16, 17](Fig 1A, right panel). In a recent study [16], Delatte et al. took the advantage of the absence of DNA 5hmdC in *Drosophila* to investigate the role of TET ortholog-promoted RNA 5hmC. They found that Tet ortholog (CG43444) mutant had smaller brain size and decreased neuroblasts in fruit fly [16], which indicated that RNA 5hmC modification plays important roles in cellular processes.

Despite the recent exciting findings about TET ortholog (CG43444)-promoted RNA 5hmC in *Drosophila*[16], little is known about the distribution of RNA 5hmC in human cells, and the regulatory mechanism of TET-promoted RNA5hmC. Interestingly, mutants of IDH1 (isocitrate dehydrogenase 1) and IDH2 (isocitrate dehydrogenase 2) gain the function of catalyzing 2-KG to oncometabolite 2-hydroxyglutarate (2-HG) [18], block the DNA oxidative activity of TETs and promote tumorigenesis [19, 20]. IDH1 and IDH2 are mutated in >75% of low grade gliomas and secondary glioblastoma multiforme (GBM), and 20% of AML [21–23]. IDH1/2 mutations identified thus far are heterozygous and produce single amino acid substitutions either at arginine 132 (R132) in IDH1 or arginine 172 (R172) in IDH2 in glioma and leukemia, or at arginine 140 (R140) in IDH2 in AML [19, 21–23]. In addition, IDH1/2 mutants gained the function of catalyzing the reduction of 2-KG to produce 2-HG to inhibit DNA oxidative activity of TETs [19, 20]. This regulatory mechanism of TETs is strongly supported by the finding that TET2 mutations are mutually exclusive with IDH1/2 mutations in AML [19, 20]. Considering the characters of IDH1/2 mutations, we hypothesized that IDH1/2 mutants could regulate the TET-promoted RNA 5hmC modification.

In this study, it was found that compared with total RNA (5hmC/C: less than 2 X 10^−7^), mRNA showed much higher 5hmC level (5hmC/C: ∼7 X 10^−6^) in human cells. It is also demonstrated that IDH1/2 mutants inhibit the RNA oxidative activity of TETs, which suggests an involvement of IDH1/2 mutations in tumorigenesis through the deregulation of RNA biology.

## Methods and Materials

### Cell culture and transfection

HEK293 and U2OS cells were maintained in Dulbecco’s modified Eagle’s medium (DMEM) containing 10% heat-inactivated FBS, 2 mM glutamine, 100 U/ml penicillin and streptomycin at 37°C under a humidified atmosphere of 5% CO_2_. Transfections were performed using lipofectamine 2000 (Thermo Fisher Scientific Inc., Waltham, MA, USA) as described in our previous studies [24, 25].

### Plasmids

pS-Flag-SBP TET1, pS-Flag-SBP TET2 and pS-Flag-SBP TET3 were gifts From Dr. Yu at University of Michigan Medical School [26]. pCDNA3B-Flag-TET1 CD, pCDNA3B-Flag-TET1 CD mut, pCDNA3B-Flag-TET2 CD, pCDNA3B-Flag-TET2 CD mut, pCDNA3B-Flag-TET3 CD and pCDNA3B-Flag-TET3 CD mut were generous gifts from Dr. Yi Zhang as described in previous study [27]. IDH1 WT, IDH1 (R132H), IDH2 WT, IDH2 (R140Q) and IDH2 (R172K) were described in the previous study [20].

### LC/MS MS analysis of DNA dC, 5mdC and 5hmdC

Genomic DNA (2 μg) was first denatured by heating at 100°C. Two units of DNase I (NEB, Ipswitch, MA, USA), 2 units of DNA degrase (Zymo Research, Irvine, CA, USA), and 2 unit of Nuclease P1 (Sigma-Aldrich, St. Louis, MO, USA) were added in a total volume of 50 μL. The resultant mixture was incubated at 37°C for 10 hours. Then one unit of alkaline phosphatase (TaKaRa, Dalian, Liaoning, China) was added to the mixture, followed by 5 hr incubation at 37°C. Complete digestion into nucleosides was confirmed by visualization of the digest on a 1% agarose gel. The reactions were diluted 2 folds, and 10 μl of the solution was injected into LC-MS/MS. The nucleosides were separated by reverse phase ultra-performance liquid chromatography on a C18 column, with mass spectrometry detection using AB SCIEX QTRAP 5500 LC/MS/MS in positive electrospray ionization mode. In brief, the digested DNA was performed using the mobile phase of 92% water (containing 0.1% formic acid) and 8.0% methanol at a flow rate of 400 μL/min. The ionspray voltage was maintained at 5500 eV. The turbo gas temperature was set at 550°C. Multiple reaction monitoring (MRM) was used to monitor the nucleoside to base ion mass transitions: m/z 228 to 112 (collision energy (CE) 15 V; declustering potential (DP) 60 V) for 2’-deoxycytidine (dC); m/z 242 to 126 (CE 18 V; DP 60 V) for 5-methyl-2’-deoxycytidine (5mdC); m/z 258 to 142 (CE 22 V; DP 60 V) for 5-hydroxymethyl-2’-deoxycytidine (5hmdC). Both Q1 and Q3 quadrupoles were maintained at unit resolution. Analyst 1.6.1 software (Applied Biosystems, Darmstadt, Germany) was used for data acquisition and processing. Linearity in ionization efficiencies were verified by analyzing dilution series of authentic standards. External calibration curves for dC, 5mdC, and 5hmdC were used to create standard curves for subsequent normalization and quantification, respectively.

### LC/MS MS analysis of RNA C, 5mC and 5hmC

Total RNA was isolated using RNeasy Mini Kit (QIAGEN, Hilden, Germany) according to the manufacturer’s instruction. Polyadenylated mRNA was purified using Dynabeads Oligo (dT)_25_ (Thermo Fisher Scientific Inc., Waltham, MA, USA) following the manufacturer’s protocol. The resultant RNA (total RNA or mRNA) were digested by 12 U nuclease P1 in 60 μl of buffer containing 25 mM NaCl, and 2.5 mM ZnCl_2_ at 37°C for overnight, followed by the addition of 1 M NH_4_HCO_3_ (3 μl) and 3 U alkaline phosphatase. After incubation at 37°C for 2 hrs, 10 μL of RNA solution was directly injected into LC-MS/MS to analyze 5hmC. Then, RNA solution was diluted 10 times and 10 μL of the diluted solution was injected into LC-MS/MS to analyze C, 5mC and 5hmC. The nucleosides were separated by reverse phase high-performance liquid chromatography on an Agilent C18 column (5-μm particle size, 150 mm x 2.1 mm), with mass spectrometry detection using AB SCIEX QTRAP 5500 LC-MS/MS in positive electrospray ionization mode. The mobile phases (delivered at 0.40 ml/min) consisted of H_2_O for A and methanol for B. An isocratic elution (80% A and 20% B) was performed at stop time of 2 min. Multiple-reaction-monitoring (MRM) mode was used to monitor analyte parent ion to product ion: m/z 274.2 to 142.1 for 5-hydroxymethyl-cytidine (5hmC) (collision energy (CE) 12 V; declustering potential (DP) 104 V; Entrance potential (EP): 13 V; collision cell exit potential (CXP): 24 V); m/z 258.1 to 126.0 for 5-methyl-cytidine (5mC) (CE 18 V; DP 60 V; EP 5 V; CXP 18V); m/z 244.1 to 112.2 for cytidine (C) (CE 22 V; DP 54 V; EP 10 V; CXP 11 V). The following instrument settings were used: column temperature: 25°C; ionspray voltage: 5500 V; turbo gas temperature: 550°C. Both Q1 and Q3 quadrupoles were maintained at unit resolution.

### LC-MS/MS analysis of 2-HG

2-hydroxyglutarate (2-HG) in metabolite extracts from cells was analyzed by LC-MS/MS. The Waters ACQUITY UPLC system and a Waters UPLC BEH C18 2.1x150 mm column (1.8 μm particles) were used in the HPLC analysis. The hydrolysates (10 μL) were injected onto the column. Formic acid (0.05%) in water and acetonitrile were used as mobile phases A and B, respectively. The elution profile was: 0–3 min, 90% A and 10% B. The mobile phase flow rate was 0.4 mL/min. Column temperature was maintained at 25°C. Mass spectrometric detection was achieved by AB SCIEX QTRAP 5500 mass spectrometer with an electrospray ionization source. Negative ionization mode, -4500V capillary voltage and 600°C source temperature were used. The optimized conditions were described as follows: declustering potential (DP), -18 V; entrance potential (EP), -4 V; collision cell exit potential (CXP), -15 V; collision energy (CE), -15 V; scan time, 200 msec. Multiple reaction monitoring (MRM) was used for the LC-MS/MS analysis. 2-HGwas detected by monitoring the quantification ion pairs of m/z 147 to 129.

### Statistical analysis

*p* values were calculated using the Student’s *t*-test. And *p* values< 0.05 were considered statistically significant.

## Results

### Triple quadrupole mass spectrometer was applied to assess DNA and RNA modifications

To assess the modification of nucleoside, triple quadrupole mass spectrometer was first applied to analyze 2’-deoxycytidine (dC), 5-methyl-2’-deoxycytidine (5mdC), 5-hydroxymethyl-2’-deoxycytidine (5hmdC), cytidine (C), 5-methylcytidine (5mC) and 5-hydroxymethylcytidine (5hmC). The nucleoside standards were separated by reverse phase ultra-performance liquid chromatography on a C18 column, with detection using AB SCIEX QTRAP 5500 LC/MS/MS in positive electrospray ionization mode. Multiple reaction monitoring (MRM) was used to monitor the nucleoside to base ion mass transitions: m/z 228 to 112 for dC; m/z 242 to 126 for 5mdC; m/z 258 to 142 for 5hmdC; m/z 244 to 112 for C; m/z 258 to 126 for 5mC; m/z 274 to 142 for 5hmC (Fig 1B). Analyst 1.6.1 software was used for data acquisition and processing. Linearity in ionization efficiencies was verified by analyzing dilution series of authentic standards. External calibration curves for DNA (dC, 5mdC and 5hmdC) and RNA (C, 5mC and 5hmC) nucleosides were used to create standard curves for subsequent normalization and quantification as noted (Fig 1C).

### Overexpression of TETs significantly increases RNA 5hmC in human HEK293 cells

**Fig 2:**
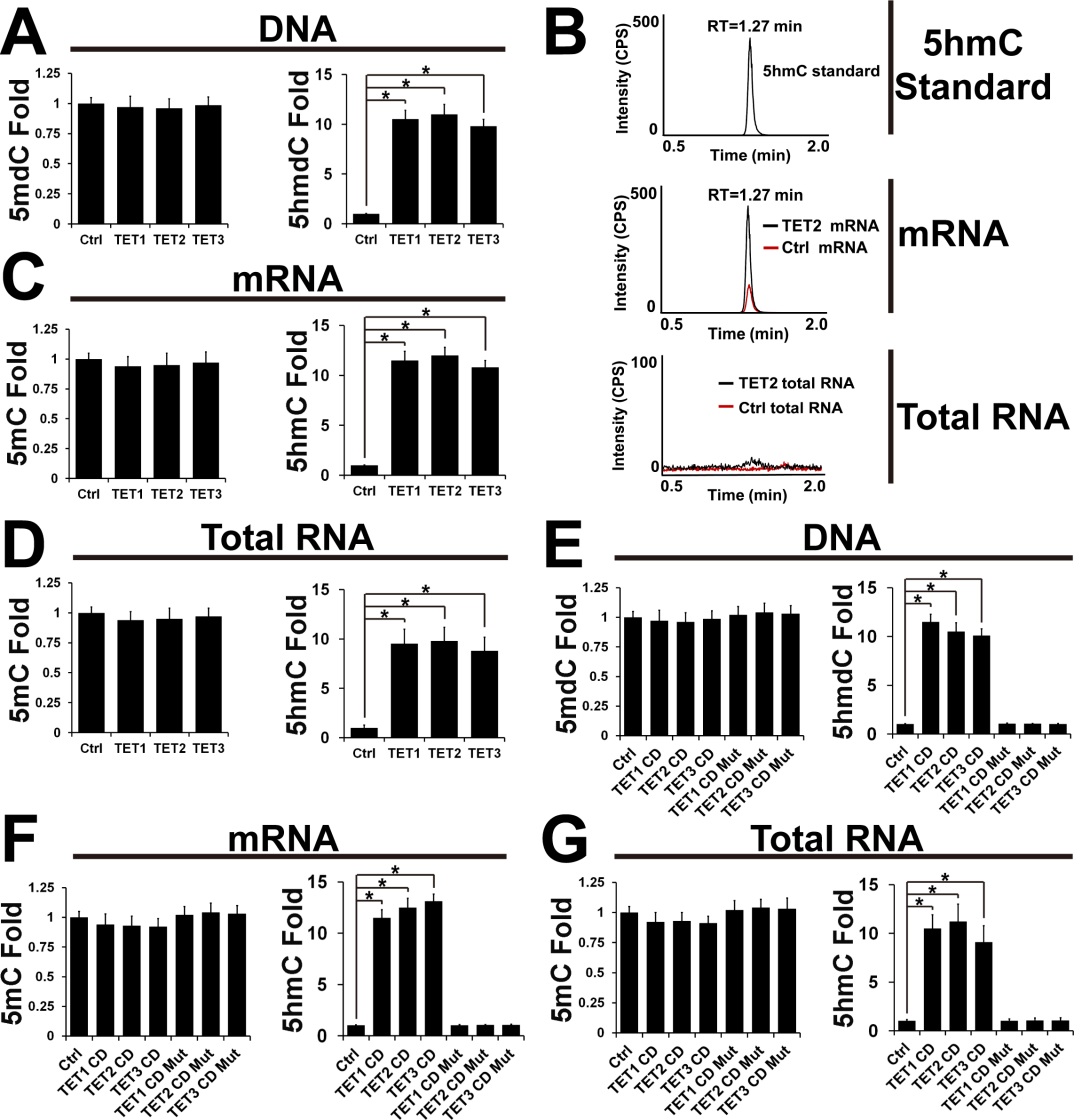
Overexpression of TETs significantly increases RNA 5hmC in human HEK293 cells. (A) HEK293 cells were transfected with pS-Flag-SBP (control), pS-Flag-SBP TET1, pS-Flag-SBP TET2 or pS-Flag-SBP TET3. After 48 hrs, DNA was extracted, digested and analyzed by mass spectrometry as described in Methods and Materials. The 5mdC level (5mdC/dC) and 5hmdC level (5hmdC/dC) were normalized. The 5mdC and 5hmdC levels in the cells transfected with pS-Flag-SBP were taken as control (Ctrl) and considered as 1. In this study, n = 3, ± SEM, and *p< 0.05 were applied unless otherwise stated. (B) The total ion chromatogram (TIC) of RNA 5hmC. Both mRNA and total RNA from (A) were extracted and digested. Resultant 6 μg of mRNA (or 6 μg of total RNA) were analyzed by LC-MS/MS as noted. TIC of RNA 5hmC represents the signal intensity normalized to RNA C level in the cells transfected with pS-Flag-SBP. RT: retention time. More details are described in Methods and Materials. (C) LC-MS/MS analysis of the mRNA (6μg) from (A). (D) LC-MS/MS analysis of the total RNA (60 μg) from (A).(E) HEK293 cells were transfected with pCDNA3 (control), pCDNA3B-Flag-TET1 CD, pCDNA3B-Flag-TET1 CD mut, pCDNA3B-Flag-TET2 CD, pCDNA3B-Flag-TET2 CD mut, pCDNA3B-Flag-TET3 CD or pCDNA3B-Flag-TET3 CD mut. After 48 hrs, DNA was extracted, digested and analyzed by LC-MS/MS. The cells transfected with pCDNA3 were taken as control (Ctrl). (F) LC-MS/MSanalysis of the mRNA from (E). (G) LC-MS/MS analysis of the total RNA from (E).

To assess the distribution of RNA 5hmC and validate the role of TETs in oxidation of RNA 5mC to 5hmC, TETs were overexpressed in HEK293 cells (S1A Fig). After 48 hrs, DNA and RNA (total RNA and mRNA) were extracted, digested and analyzed by mass spectrometry as described in Methods and Materials. Consistent with the previous studies [2, 27], overexpression of TETs enhanced the DNA 5hmdC (Fig 2A). After the normalization of 5hmC according to C, it was found that 5hmC in total RNA was almost undetectable (5hmC/C: less than2 X 10^−7^) in HEK293 cells, whereas 5hmC in mRNA was present at an appreciable level (5hmC/C: ∼7 X 10^−6^) (Fig 2B). As shown in Fig 2B, there were only tiny and distorted TIC peaks for 5hmC in total RNA samples, whereas there were sharp TIC peaks in mRNA samples. In another word, compared with total RNA, mRNA was highly modified by 5hmC in human cells, which suggested that 5hmC might be involved in mRNA splicing, transporting and translation. Further study indicated that overexpression of full length TETs significantly increases both mRNA and total RNA 5hmC levels (5hmC/C) (Fig 2C and 2D). To confirm these results, wild-type catalytic domain (CD) and catalytic dead mutant (CD mut) of TET1 (1367–2039), TET2 (916–1921) or TET3 (697–1668) were transfected into HEK293 cells (S1B Fig). The catalytic dead mutant (CD mut) forms of TETs were taken as negative controls [27]. Resultant DNA and RNA were extracted, digested and analyzed by mass spectrometry. Consistent with the previous study [27], the catalytic domain (CD) forms of TETs, but not their catalytic dead mutant (CD mut), promoted DNA 5hmdC modification (Fig 2E). And mass spectrometry analysis indicated that TET CDs significantly enhanced both mRNA and total RNA 5hmC levels as expected (Fig 2F and 2G).

### IDH1/2 mutants inhibit TET-promoted oxidation of RNA 5mC to 5hmC

**Fig 3:**
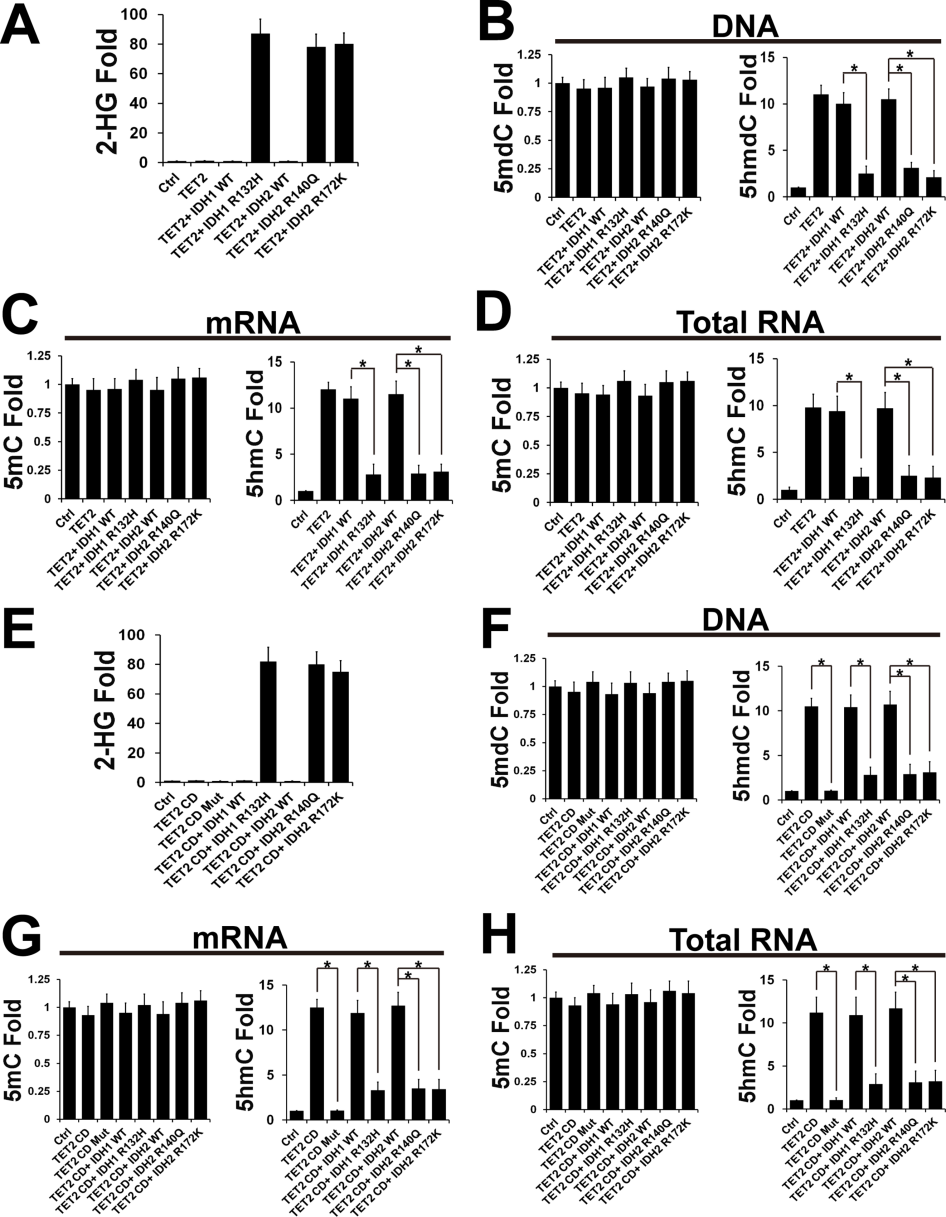
IDH1/2 mutants inhibit TET-promoted oxidation of RNA 5mC to 5hmC. (A) HEK293 cells were transfected with/without pS-Flag-SBP (control), pS-Flag-SBP TET2, IDH1 WT, IDH1 (R132H), IDH2 WT, IDH2 (R140Q) and/or IDH2 (R172K) as noted. After 48 hrs, 2-HG was extracted and analyzed by mass spectrometry. The cells transfected with pS-Flag-SBP were taken as control (Ctrl). (B) LC-MS/MS analysis of the DNA from (A). (C) LC-MS/MS analysis of the mRNA from (A). (D) LC-MS/MS analysis of the total RNA from (A). (E)HEK293 cells were transfected with/without pCDNA3, pCDNA3B-Flag-TET2 CD, pCDNA3B-Flag-TET2 CD mut, IDH1 WT, IDH1 (R132H), IDH2 WT, IDH2 (R140Q) and/or IDH2 (R172K) as noted. After 48 hrs, 2-HG was extracted and analyzed by mass spectrometry. The cells transfected with pCDNA3 were taken as control (Ctrl). (F) LC-MS/MS analysis of the DNA from (E). (G) LC-MS/MS analysis of the mRNA from (E). (H) LC-MS/MS analysis of the total RNA from (E).

To validate the role of IDH1/2 mutants in TET-promoted RNA oxidation, TET2 with/without IDH1/2 wts or mutants as noted were transfected into HEK293 (S1C Fig)or U2OS cells. After 48 hrs, 2-hydroxyglutarate (2-HG), DNA and RNA were extracted and analyzed by mass spectrometry as described in Methods and Materials. Consistent with the previous studies [2, 27], mass spectrometry analysis demonstrated that IDH1/2 mutants enhanced 2-HG and inhibited the DNA oxidative activity of TETs (Fig 3A and 3B). Immunofluorescence of 5hmdC also confirmed that overexpression of IDH1 mutant (R132H) inhibited TET-promoted DNA 5hmdC in U2OS cells (S1D Fig). Mass spectrameter analysis indicated that IDH1/2 mutants also inhibited the RNA oxidative activity of TETs (Fig 3C and 3D). To confirm these results, TET2 CD with/without IDH1/2 wts or mutants as noted was transfected into HEK293 cells (S1E Fig). Resultant 2-HG, DNA and RNA were extracted and analyzed by mass spectrometry. Consistent with the results from full length TET2, IDH1/2 mutants also inhibited TET CD-promoted oxidation of DNA, mRNA and total RNA (Fig 3E–3H).

## Discussion

Although it is generally believed that TETs play roles in cellular processes through DNA oxidation, recent studies indicated that TET ortholog (CG43444) and TETs are also able to oxidate RNA 5mC to 5hmC [16, 17, 28]. However, little is known about the distribution of RNA 5hmC in human cells. In this study, It was found that 5hmC of total RNA was almost undetectable (5hmC/C: less than 2 X 10^−7^) in human cells, whereas 5hmC of mRNA was present at an appreciable level (5hmC/C: ∼7 X 10^−6^). The enrichment of 5hmC in mRNA and the involvement of TETs in mRNA oxidation suggest that the roles of TETs are not restricted to the transcriptional regulation at the DNA level, but highly likely can be extended to the translational regulation at the mRNA level. Further studies are needed to define the site-specific localization of 5hmC in human mRNA, and the function of 5hmC modification in RNA biology. Due to the critical roles of TETs in various physical and pathological processes, the potential functions of TETs in RNA biology should also be evaluated.

In addition, despite the recent exciting findings about TET ortholog (CG43444)-promoted RNA 5hmC [16], little is known about the regulatory mechanism of TET-promoted RNA 5hmC. Previous studies have indicated that IDH1/2 mutants gain the function of catalyzing 2-KG to 2-HG [18], block the DNA oxidative activity of TETs and promote tumorigenesis [19, 20]. Here, we demonstrate that IDH1/2 mutants can inhibit TET-promoted oxidation of RNA 5mC to 5hmC. This IDH1/2 mutant-promoted inhibition of mRNA 5hmC suggests that IDH1/2 mutants are potentially involved in RNA biology including mRNA splicing, transporting, translation and degradation. As mentioned above, IDH1 and IDH2 are mutated in >75% of low grade gliomas and secondary glioblastoma multiforme (GBM), and 20% of AML [21–23]. Given the central roles of IDH1/2 mutations in tumorigenesis [19, 20], further studies are needed to evaluate the role of RNA 5hmC in the tumorigenesis.

However two limitations of this study should be noticed. First, we focused on the overexpression of TETs in this study, due to the abundance of 5hmC in RNA. Second, only HEK293 cells were applied to evaluate the role of IDH1/2 mutants in TET-promoted oxidation of RNA 5mC to 5hmC. Notwithstanding these two limitations, this study clearly demonstrates the enrichment of 5hmC in mRNA, and the ability of IDH1/2 mutants to inhibit the RNA oxidative activity of TETs.

Collectively, we describe the mRNA enrichment of 5hmC in human cells, which suggests 5hmC might be involved in RNA biology including mRNA splicing, transporting, translation and degradation. Furthermore, we also demonstrate that IDH1/2 mutants can inhibit TET-promoted RNA 5hmC modification, which suggests an involvement of IDH1/2 mutations in tumorigenesis through the deregulation of RNA biology.

## Supporting Information

S1 Fig Immunoblotting and immunofluorescence, related to Fig 2 and Fig 3. (TIF)

S1 Methods and Materials Methods and Materials, related to immunoblotting and immunofluorescence. (DOCX)
