# Supplementary figures and images for: IDH1/2 Mutants Inhibit TET-Promoted Oxidation of RNA 5mC to 5hmC
Source: PLoS One. 2016 Aug 22;11(8):e0161261. doi: 10.1371/journal.pone.0161261 (PMC4993491; doi:10.1371/journal.pone.0161261)

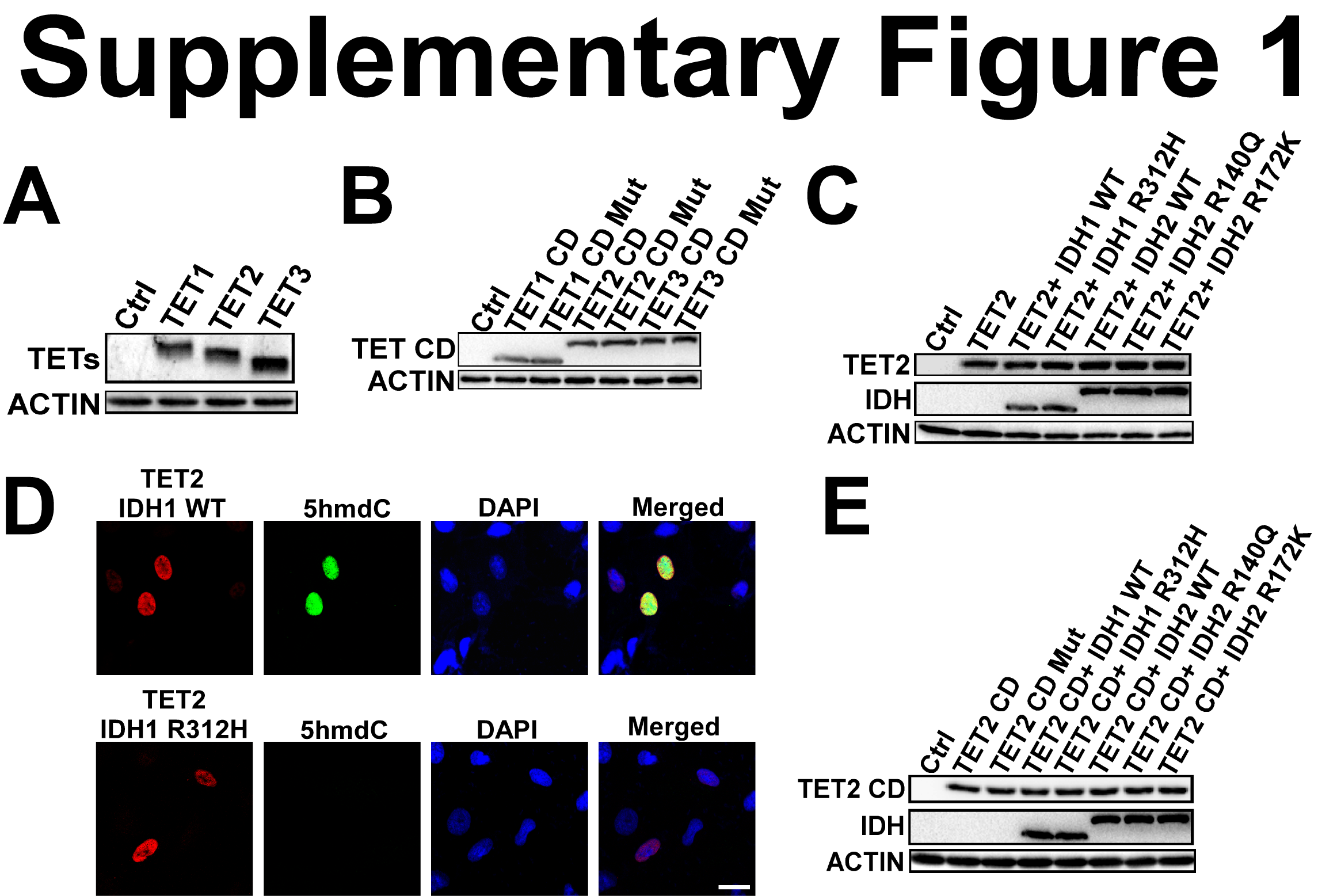

Supplement: S1 Fig — (TIF) [file pone.0161261.s001.tif]
