# Supplementary material for: IDH1/2 Mutants Inhibit TET-Promoted Oxidation of RNA 5mC to 5hmC
Source: PLoS One. 2016 Aug 22;11(8):e0161261. doi: 10.1371/journal.pone.0161261 (PMC4993491; doi:10.1371/journal.pone.0161261)
Supplement: S1 Methods and Materials — (DOCX) [file pone.0161261.s002.docx]

**Supporting Information**

**S1 Methods and Materials**

**Immunoblotting**

The cells were harvested in RIPA buffer (1X PBS, 1% NP40, 0.1% SDS, 0.5% sodium deoxycholate, 0.1 mg/ml PMSF and 1 mM sodium orthovanadate). Resultant lysates were resolved by SDS-PAGE, transferred to nitrocellulose membrane, blocked in 5% nonfat milk, and blotted with the appropriate antibody.

**Immunofluorescence**

Cells were first plated on coverslips. After transfected with the plasmids, the cells were washed with PBS for 3 times and fixed with 4% formaldehyde in PBS for 10 min. After being washed with PBS for 3 X 10 min, the cells were permeabilized with 0.5% Triton-X 100 in PBS for 10 min. The permeabilized cells were denatured with 2N HCl for 15 min and neutralized with 100 mM Tris-HCl (pH 8.5) for 10 min. After blocking, the cells were incubated with anti-5-hydroxymethylcytosine (5-hmdC) antibody (Active Motif, Carlsbad, CA, USA) for 1.5 hrs. Then the cells were washed with 1% NGS and incubated with anti-Flag antibody for 1.5 hrs. After being washed with PBS for 3 X 10 min, the coverslips were mounted with a drop of mounting medium, sealed with clear nail polish, and visualized using Leica TCS SP5 (DMI6000CS) scanning laser confocal microscope.

**S1 Fig. Figure Legends**

**S1 Fig. Immunoblotting and immunofluorescence, related to Fig 2 and Fig 3.** (A) Immunoblotting of the total cell lysates from Fig 2A using anti-Flag and anti-ACTIN antibodies (Cell Signaling, Beverly, MA, USA). (B) Immunoblotting of the total cell lysates from Fig 2E. (C) Immunoblotting of the total cell lysates from Fig 3A. (D) Immunofluorescence of U2OS cells transfected with the plasmids as noted. Scale bar = 10μm. (E) Immunoblotting of the total cell lysates from Fig 3E.
